# WIP1 deficiency inhibits HTLV-1 Tax oncogenesis: novel therapeutic prospects for treatment of ATL?

**DOI:** 10.1186/1742-4690-9-115

**Published:** 2012-12-21

**Authors:** Nicolas Gillet, Alexandre Carpentier, Pierre-Yves Barez, Luc Willems

**Affiliations:** 1 Molecular and Cellular Epigenetics, GIGA, University of Liège, Liège, Belgium; 2 Molecular biology, GxABT, University of Liège, Gembloux, Belgium

**Keywords:** HTLV-1, Tax, HBZ, p53, Wip1, PPM1D, MDM2, DNA damage response, Genomic stress, ATM, Chk2

## Abstract

Attenuation of p53 activity appears to be a major step in Human T-lymphotropic virus type 1 (HTLV-1) Tax transformation. However, p53 genomic mutations are late and rather infrequent events in HTLV-1 induced Adult T cell leukemia (ATL). The paper by Zane *et al*. shows that a mediator of p53 activity, Wild-type p53-induced phosphatase 1 (Wip1), contributes to Tax-induced oncogenesis in a mouse model. Wip1 may therefore be a novel target for therapeutic approaches.

## Background

**Figure 1:**
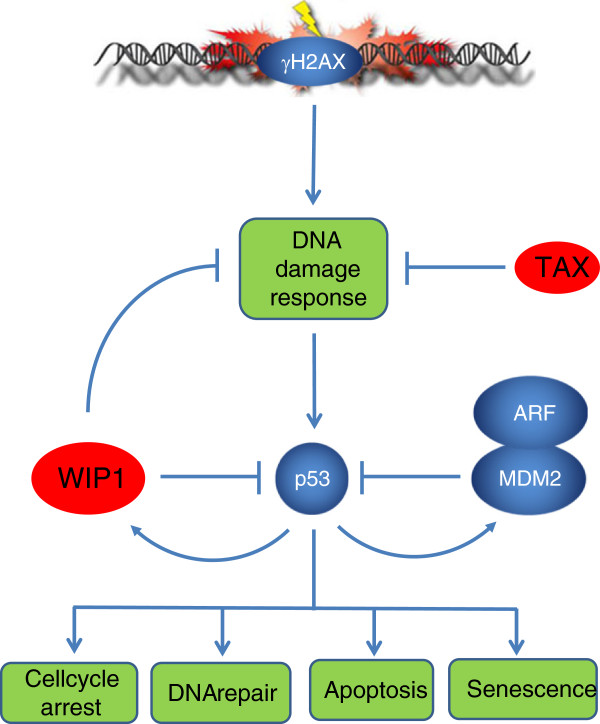
**Schematic view of the Tax/p53/Wip1/MDM2/ARF interplay.** Double strands breaks (DSB) induced by Tax-driven genomic stress are frequent in ATL cells. Recognition of these DSB by γH2AX initiates the DNA damage response (DDR) and signals to the p53 tumor suppressor that arrests the cell cycle, allows DNA repair, and induces apoptosis or senescence. p53 activity is controlled by MDM2/ARF and Wip1. MDM2 is an E3 ubiquitin ligase that degrades p53 and is a first arm of a p53-negative feedback loop. The second mechanism is created by Wip1 that inhibits the DDR through dephosphorylation of ATM Ser1981 and CHK2 Thr68.

Human T-lymphotropic virus type 1 (HTLV-1) is a δ−retrovirus that infects about 25 million people worldwide [1,2]. HTLV-1 causes Adult T-cell leukemia-lymphoma (ATLL) and a neurodegenerative disease called HTLV-induced myelopathy - tropical spastic paraparesis (HAM/TSP). Only a minority of infected subjects (~4%) will develop either ATL or HAM/TSP usually after several decades of latency. Although the mechanisms are still incompletely understood, experimental evidence shows that two factors, Tax and HBZ, are major players in viral replication and oncogenesis [3-5]. The HBZ gene is transcribed from the proviral complementary strand initiating from the 3^′^-LTR promoter. HBZ activates JunD transcription and TGFβ signaling but inhibits the canonical NF-κB pathway and 5^′^-LTR-directed transcription [4]. Unexpectedly, HBZ RNA was reported as sufficient to promote T cell proliferation. The other HTLV-1 oncogene, Tax, is a transcriptional activator of viral expression that modulates major intracellular signaling pathways (NF-κB, CREB and AP1) [1]. Tax immortalizes cells *in vitro* and induces tumors in transgenic mouse models. Tax stimulates S phase progression by accelerating early firing of late replication origins [6]. This process thus permits faster cell proliferation but also engenders DNA strand breaks due to unscheduled replication timing. This mechanism creates an oncogenic stress that triggers the DNA damage response (DDR) [7]. Concomitantly, Tax also attenuates the DDR by interacting with different components of the p53 pathway such as Chk2 [8] (Figure 1). The p53 tumor suppressor controls cell cycle arrest allowing DNA repair and, if damage cannot be repaired, induces apoptosis or senescence. p53 is thus a major player that directs cell fate upon infection by HTLV-1, as observed for a broad variety of other viruses. The mechanisms by which Tax attenuates p53 activity are still debated, but clearly depend on the cell types and the experimental settings. Nonetheless, it is evident that the inactivation of p53 by gene mutation is less frequent in ATL compared to other human neoplasms (17-42% versus 60%) [9]. Together, these observations enlighten the importance of Tax-mediated inactivation of p53 in ATL.

### Wild-type p53-induced phosphatase 1

In the paper by Zane *et al*., the predicted role of p53 was first validated in a transgenic mouse model, confirming previous reports. By crossing Tax-transgenic and p53 knock-out mice, they show that tumor-free survival is significantly shortened in a p53^−/−^ background. The authors next evaluated the role of Wip1 (Wild-type p53-induced phosphatase 1), a regulator of p53. Interestingly, Wip1 deficiency reduces Tax induced tumorigenesis in Wip1^−/−^ and Wip1^+/−^ mice. Consistent with their inhibitory activity, transient expression of Tax and Wip1 reduced p53 transcriptional activity in reporter assays. Although the mechanisms still need to be further characterized, a plausible model is that Tax and Wip1 cooperate in tumorigenesis via p53 inactivation. The paper by Zane *et al*. thus extends previous observations showing resistance to transformation by other oncogenes such as Ras, Myc, E1A and Erbb2 in PPM1D deficient cells [10].

Wip1 is a PP2C family serine/threonine phosphatase that inhibits the function of several tumor suppressor pathways, including ATM, CHK2, p38MAPK and p53 [10]. PPM1D (protein phosphatase, Mg2+/Mn2+ dependent, 1D), the gene encoding Wip1, is aberrantly amplified in different types of human primary cancers. Conversely, deletion of PPM1D in mice decreases tumorigenesis. In breast cancers, p53 mutations are frequent, but tumors with PPM1D amplification rarely harbor p53 mutations. One explanation is that Wip1 promotes tumor formation through its ability to inhibit p53 tumor suppressor function directly or indirectly, thus reducing selective pressure for p53 mutations during the progression of cancer.

The paper by Zane *et al*. is important because it reveals the potential oncogenic role of Wip1 in Tax-mediated oncogenesis in a model of ATL. A series of open questions remain regarding the biological relevance of Wip1 in patients and the mechanisms involved. First, it is currently unknown if gene amplification of PPM1D occur in ATL as observed in other types of cancers. Like p53 deletions [9,11], PPM1D genomic amplifications usually appear at late stages of tumorigenesis. Alternatively, it is possible that expression and/or activity of Wip1 are increased in the absence of genomic alteration. The PPM1D promoter contains at least two types of transcription binding sites: one for p53 that creates a negative feedback loop (Figure 1) and another for c-Jun [10]. Current data show that Tax, despite its ability to stimulate AP1, does not activate PPM1D expression in reporter assays. Moreover, Wip1 mRNA expression is not increased in Tax-positive cells. This observation needs to be further validated at the protein level using a larger number of HTLV-1 infected cell lines as well as in patients’ samples. Confocal microscopy indicates that Tax and Wip1 colocalize in the nucleus. Further experiments will be required to determine direct or indirect binding between Wip1 and Tax. However, interactomic and proteomic analyzes currently suggest that both proteins do not physically interact (JC Twizere and OJ Semmes, personal communications).

In the context of HTLV-1 associated oncogenesis, it will be interesting to assess the role of other viral oncogenes like HBZ in the Tax/Wip1/p53 interplay. As an inhibitor of AP1 activity, HBZ might interfere with Wip1 expression. Combined crosses between Tax, Wip1, p53 and HBZ transgenic and knock-out mice could address this question. In this context, it is noteworthy that deletion of p14(ARF), a MDM2 modulator (Figure 1), accelerates osteosarcoma formation further supporting the role of p53 regulation in Tax-induced oncogenesis [12]. ARF is a sensor of hyperproliferative signals such as those from the Ras and Myc oncoproteins. In response to oncogenic stress, ARF causes cell-cycle arrest in G1 and G2/M and is associated with increased p53 and p21 expression. ARF mediates cell-cycle arrest by directly binding to MDM2 and sequesters it in the nucleolus. Sequestration of MDM2 stabilizes and activates p53 which then blocks cellular proliferation. Although ARF does not appear to be aberrantly expressed in ATLL cells [9], acceleration of Tax-induced tumor formation in ARF-deficient mice further supports the central role exerted by p53.

**Figure 2:**
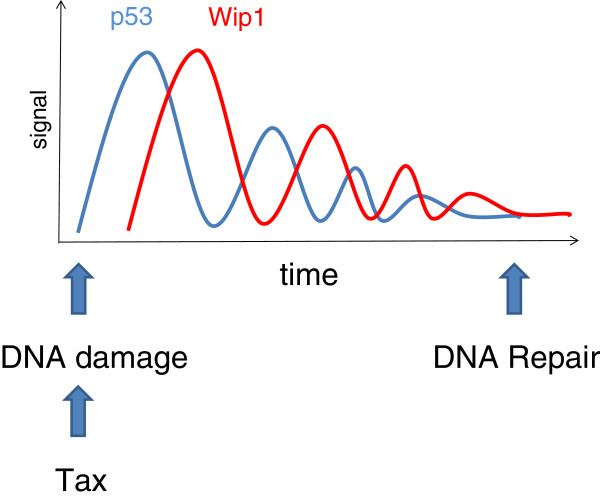
**Importance of Wip1 in modulating an oscillatory negative feedback loop within the p53 response pathway.** Tax-induced DNA damage initiates a cascade that stimulates p53 expression. p53 then upregulates Wip1 creating a negative feedback loop. If DNA damage has not been completely repaired during the first pulse, the cycle repeats with a lower intensity. This mechanism generates a series of p53 and Wip1 pulses in an oscillating pattern that attenuates until the DNA damage is repaired.

Of particular interest is the Wip1-dependent regulation of ATM-CHK2 signaling on the dynamics of p53-dependent response to DNA damage [10]. In fact, recruitment of γH2AX to double strand breaks initiates a cascade that produces different levels of p53 depending on the level of damage. This mechanism also initiates a series of p53 pulses in a repetitive pattern that shapes p53 protein level dynamics in an oscillating manner due to positive and negative feedback loops (Figure 2). If DNA damage has not been completely repaired during the first pulse, the cycle repeats with a lower intensity. MDM2 and Wip1 are two essential components of this feedback loop. Upregulation of p53 during the first pulse activates transcription of MDM2 and Wip1 which, in turn, can feedback negatively to p53 and ATM-Chk2, respectively. Disequilibrium in this intricate network could lead to incomplete DNA repair.

What are the outcomes of these observations in terms of therapy of ATL? First, Tax-repressed p53 function in HTLV-1-transformed cells is druggable and can be restored by treatment with 9-aminoacridine in a setting absent for p53 genomic alterations [13]. Secondly, arsenic trioxide, which has been recently proposed to prevent relapse of ATL lymphoma patients [14], augments Chk2/p53-mediated apoptosis by inhibiting Wip1 [15]. Thirdly, Wip1 can directly be targeted by specific inhibitors such as compound M (mercury, [4-Aminophenyl] [6-thioguanosinato-N7,S6]), CCT007093 ([2E,5E]-2,5-Bis[2-thienylmethylene]-cyclopentanone) or thioether cyclic phosphopeptide c (MpSIpYVA) [10]. Alternatively, phosphorothioate antisense oligonucleotides targeting PPM1 and specifically disrupting the binding of ATM–Wip1 or CHK2–Wip1 may be instrumental providing that delivery issues are solved.

## Conclusion

In summary, the paper by Zane *et al*. adds a new piece in the complex puzzle of Tax-induced oncogenesis and reveals a potential new mechanism of p53 attenuation during HTLV-1 pathogenesis opening interesting prospects for therapy.

## Competing interests

The authors declare no competing financial interests.

## Authors’ contributions

NG, AC, PYB and LW wrote and approved the manuscript. All authors read and approved the final manuscript.

## References

[B1] Boxus M Willems L Mechanisms of HTLV-1 persistence and transformation Br J Cancer 2009 101 9 1497 1501 10.1038/sj.bjc.6605345 19861996 PMC2778510

[B2] Martin F Bangham CR Ciminale V Lairmore MD Murphy EL Switzer WM Mahieux R Conference highlights of the 15th international conference on human retrovirology: HTLV and related retroviruses, 4–8 june 2011, leuven, gembloux, Belgium Retrovirology 2011 8 86 10.1186/1742-4690-8-86 22035054 PMC3223150

[B3] Twizere JC Kruys V Lefebvre L Vanderplasschen A Collete D Debacq C Lai WS Jauniaux JC Bernstein LR Semmes OJ Interaction of retroviral Tax oncoproteins with tristetraprolin and regulation of tumor necrosis factor-alpha expression J Nat Cancer Institute 2003 95 24 1846 1859 10.1093/jnci/djg118 14679154

[B4] Zhao T Matsuoka M HBZ and its roles in HTLV-1 oncogenesis Front Microbiol 2012 3 247 22787458 10.3389/fmicb.2012.00247 PMC3391691

[B5] Hagiya K Yasunaga J Satou Y Ohshima K Matsuoka M ATF3, an HTLV-1 bZip factor binding protein, promotes proliferation of adult T-cell leukemia cells Retrovirology 2011 8 19 10.1186/1742-4690-8-19 21414204 PMC3068935

[B6] Boxus M Twizere JC Legros S Kettmann R Willems L Interaction of HTLV-1 Tax with minichromosome maintenance proteins accelerates the replication timing program Blood 2012 119 1 151 160 10.1182/blood-2011-05-356790 22058115

[B7] Boxus M Willems L How the DNA damage response determines the fate of HTLV-1 Tax-expressing cells Retrovirology 2012 9 2 10.1186/1742-4690-9-2 22221708 PMC3283471

[B8] Durkin SS Guo X Fryrear KA Mihaylova VT Gupta SK Belgnaoui SM Haoudi A Kupfer GM Semmes OJ HTLV-1 Tax oncoprotein subverts the cellular DNA damage response via binding to DNA-dependent protein kinase J Biol Chem 2008 283 52 36311 36320 10.1074/jbc.M804931200 18957425 PMC2605996

[B9] Takemoto S Trovato R Cereseto A Nicot C Kislyakova T Casareto L Waldmann T Torelli G Franchini G p53 stabilization and functional impairment in the absence of genetic mutation or the alteration of the p14(ARF)-MDM2 loop in ex vivo and cultured adult T-cell leukemia/lymphoma cells Blood 2000 95 12 3939 3944 10845931

[B10] Le Guezennec X Bulavin DV WIP1 phosphatase at the crossroads of cancer and aging Trends biochem sci 2010 35 2 109 114 10.1016/j.tibs.2009.09.005 19879149

[B11] Dequiedt F Kettmann R Burny A Willems L Mutations in the p53 tumor-suppressor gene are frequently associated with bovine leukemia virus-induced leukemogenesis in cattle but not in sheep Virology 1995 209 2 676 683 10.1006/viro.1995.1303 7778302

[B12] Rauch DA Hurchla MA Harding JC Deng H Shea LK Eagleton MC Niewiesk S Lairmore MD Piwnica-Worms D Rosol TJ The ARF tumor suppressor regulates bone remodeling and osteosarcoma development in mice PLoS One 2010 5 12 e15755 10.1371/journal.pone.0015755 21209895 PMC3012707

[B13] Jung KJ Dasgupta A Huang K Jeong SJ Pise-Masison C Gurova KV Brady JN Small-molecule inhibitor which reactivates p53 in human T-cell leukemia virus type 1-transformed cells J Virol 2008 82 17 8537 8547 10.1128/JVI.00690-08 18550670 PMC2519633

[B14] Bazarbachi A Suarez F Fields P Hermine O How I treat adult T-cell leukemia/lymphoma Blood 2011 118 7 1736 1745 10.1182/blood-2011-03-345702 21673346

[B15] Yoda A Toyoshima K Watanabe Y Onishi N Hazaka Y Tsukuda Y Tsukada J Kondo T Tanaka Y Minami Y Arsenic trioxide augments Chk2/p53-mediated apoptosis by inhibiting oncogenic Wip1 phosphatase J Biol Chem 2008 283 27 18969 18979 10.1074/jbc.M800560200 18482988

